# A deforestation-induced tipping point for the South American monsoon system

**DOI:** 10.1038/srep41489

**Published:** 2017-01-25

**Authors:** Niklas Boers, Norbert Marwan, Henrique M. J. Barbosa, Jürgen Kurths

**Affiliations:** 1 Ecole Normale Supérieure, Geosciences Department and Laboratoire de Météorologie Dynamique, Paris, F-75230, France; 2 Potsdam Institute for Climate Impact Research, Potsdam, 14473, Germany; 3 University of São Paulo, Institute of Physics, São Paulo, 05508-090, Brazil; 4 Humboldt University, Department of Physics, Berlin, 12489, Germany; 5 Nizhny Novgorod State University, Department of Control Theory, Nizhny Novgorod, 603950, Russia; 6 University of Aberdeen, Institute for Complex Systems and Mathematical Biology, Aberdeen, AB24 3UE, United Kingdom

## Abstract

The Amazon rainforest has been proposed as a tipping element of the earth system, with the possibility of a dieback of the entire ecosystem due to deforestation only of parts of the rainforest. Possible physical mechanisms behind such a transition are still subject to ongoing debates. Here, we use a specifically designed model to analyse the nonlinear couplings between the Amazon rainforest and the atmospheric moisture transport from the Atlantic to the South American continent. These couplings are associated with a westward cascade of precipitation and evapotranspiration across the Amazon. We investigate impacts of deforestation on the South American monsoonal circulation with particular focus on a previously neglected positive feedback related to condensational latent heating over the rainforest, which strongly enhances atmospheric moisture inflow from the Atlantic. Our results indicate the existence of a tipping point. In our model setup, crossing the tipping point causes precipitation reductions of up to 40% in non-deforested parts of the western Amazon and regions further downstream. The responsible mechanism is the breakdown of the aforementioned feedback, which occurs when deforestation reduces transpiration to a point where the available atmospheric moisture does not suffice anymore to release the latent heat needed to maintain the feedback.

Many studies have suggested that the Amazon rainforest may be a potential tipping element of the earth system^1^,^2^. Results of several coupled global climate models have indicated the possibility of a future dieback of the rainforest under global warming scenarios^3^,^4^, but also ongoing deforestation has been discussed as a possible cause of a regime shift of the ecosystem^5^,^6^,^7^,^8^,^9^,^10^,^11^,^12^. Here, we propose a model of the nonlinear couplings between the atmospheric moisture transport over South America and the Amazon rainforest, which are associated with a westward cascade of precipitation and evapotranspiration. Impacts of ongoing deforestation on the South American low-level circulation will be analyzed with particular focus on a positive atmospheric feedback induced by condensational latent heat release over the Amazon^13^,^14^,^15^, which is neglected in most studies investigating the consequences of deforestation on the resilience of the Amazonian rainforest.

Rainfall in vast parts of South America critically depends on the atmospheric moisture inflow from the tropical Atlantic ocean. After crossing the Amazon basin, these moist low-level winds are blocked by the Andes mountains to the west and channelled southwards, forming a low-level jet from the western Amazon basin to the subtropics, for which it is the most important moisture source^16^.

Due to the release of latent heat (*LH*), precipitation over tropical South America strengthens the atmospheric heating gradient between the Atlantic ocean and the continent, and thereby enhances the low-level atmospheric inflow into the Amazon basin. This heating gradient can be estimated to enhance the easterly inflow into South America by a factor between 2 and 3 during the monsoon season (December–February)^13^,^14^,^15^. The Amazon rainforest’s evapotranspiration (*E*) recharges the low-level atmosphere’s moisture content, resulting in additional moisture being available for precipitation (*P*) further downstream of the westward flow. In turn, high *P* rates and the associated condensational heating are crucial for the existence of the rainforest itself, and thus for maintaining high *E* rates in the long term. Due to these feedback mechanisms, widespread deforestation does not only impact the ecosystem locally, but may cause nonlinear responses of the atmospheric circulation regime, and thereby impact climate in other regions as well^1^. In particular, the easterly low-level flow across the Amazon basin will cause the impacts of deforestation in terms of available moisture and *LH* release to cascade westward to yet undisturbed parts of the rainforest and further downstream toward the subtropics. For example, a recent study estimates that as much as 70% of *P* in the La Plata basin, a region with extensive agricultural activity^17^, originate from *E* in the Amazon basin^18^.

Most existing studies investigate the impacts of deforestation in the Amazon basin by comparing model results obtained from scenarios with intact rainforest to results obtained from scenarios where the rainforest is completely removed^5^,^8^,^19^,^20^. While this is certainly useful for assessing the climatological relevance of the Amazonian ecosystem, it is less helpful to understand the specific ways in which ongoing deforestation will successively affect the biosphere-atmosphere couplings in terms of moisture recycling and condensational *LH* release. In contrast, two recent studies^9^,^21^ based on general circulation models (GCMs) analyse impacts of successive deforestation on *P*, and find that *P* decreases weakly nonlinearly as deforestation proceeds. However, the first^9^ only investigates impacts on *P* over the eastern Amazon basin, although the cascading effects of deforestation in the eastern Amazon can be expected to be more severe in the western regions, further downstream of the low-level flow. The latter^21^ does not analyse the atmospheric mechanisms causing the nonlinearities. Furthermore, such GCM-based studies are based on single realisations of the multitude of possible parameters used in the GCM equations. The huge uncertainties associated with these parameter choices can in general hardly be estimated in a rigorous way^22^. For example, projections of the future fate of the Amazon vary substantially between different GCMs, and even from one version of a single GCM to the next^23^. The sensitivity of *P* over the Amazon basin against small variations of the relevant parameters, such as deforestation-induced changes in surface net radiation and the heating gradient between ocean and land, cannot be investigated along those lines. However, knowledge of this sensitivity is essential for identifying a possible tipping point in the precipitation regime. Existing conceptual approaches^24^,^25^ have modelled deforestation in a single box, and are therefore not capable of analysing the cascading impacts of deforestation.

In order to study the cascading, nonlinear effects of deforestation, we construct a nonlinear model of the moisture transport along a trajectory covering the entire Amazon basin. This approach allows for an isolation of the specific relationship between a deforestation-induced decrease of total surface heat flux (including, in particular, the decrease of *E*), and the positive feedback associated with atmospheric *LH* release. Furthermore, such a conceptual model is essential to gain a physical understanding of the involved dynamical processes, and to be able to investigate the consequences of successive deforestation for wide ranges of the relevant parameters. We will in the following focus on the monsoon season (December–February) in order to stay in a conservative setting, since impacts of deforestation can be assumed to be more severe during the dry season.

The underlying equations are dictated by the conservation of water in the hydrological cycle:


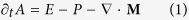



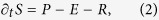


where *A* and *S* denote total moisture content in the atmosphere and soil, respectively, *E* denotes evapotranspiration, *P* is precipitation, and *R* is river runoff. In addition, 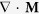 denotes the divergence of vertically integrated atmospheric moisture flow: at each atmospheric layer *λ*, this moisture flow is defined as 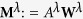, where **W**^*λ*^ denotes the wind speed. The variables *P, E* and *R* will be modelled as effective functions of *A* and *S*, respectively (see Figs S1, S2, and S3). Wind speeds **W** are in our model comprised of a trade wind component and a component representing the amplification of the wind speeds due to the gradient in atmospheric heating between the tropical Atlantic ocean and the Amazon basin (Fig. 1): **W** = **W**^*trade*^ + **W**^*H*^. The latter setting introduces the nonlinearity to the model, since **W**^*H*^ depends on atmospheric condensation and hence *A* itself. The model equations are integrated along a sequence of 100 spatial boxes following the climatological trajectory of the low-level winds from the mouth of the Amazon river to the western boundary of the basin (Fig. 1).

For the specific formulation of the model, as well as details concerning the employed data sets and simulations, we refer to the methods section below. The results presented in the following will show that the positive feedback associated with atmospheric *LH* release is indeed the crucial mechanism behind the high moisture inflow from ocean to land, and that there exists a threshold for the extents of deforestation, beyond which this mechanism can no longer be maintained.

## Results

### Model evaluation

In order to validate the proposed nonlinear model, we compare the modelled development of the six observables *A, S, E, P, R*, and *W* (Fig. 2), as well as the different components of atmospheric heating (Fig. S4), with the corresponding values obtained from the ERA Interim reanalysis^26^. We find that the modelled observables are in very good agreement with the corresponding reanalysis data. Only for the western part of the trajectory, we obtain higher values of *A* and, therefore, *P* and *R* than suggested by ERA Interim. However, a comparison with the more reliable data provided by the gauge-calibrated satellite product TRMM 3B42 V7^27^ indicates that the ERA Interim reanalysis underestimates *P* in western tropical South America, and that our model results are in fact closer to the satellite-derived observations than ERA Interim (Fig. S5). Values shown for ERA Interim and TRMM 3B42 are multi-year averages of the monsoon seasons (December–February) during the time spans 1979–2014 and 1998–2014, respectively.

As a further corroboration of our model, we find that 35% of atmospheric moisture content at the final box (#100) stems from *E* along the trajectory, which compares well with a recent estimate of continental precipitation recycling ratios^18^.

### Consequences of deforestation

We investigate the impacts of deforestation on *P* downstream of the low-level flow for a range of scenarios, corresponding to the range of possible amplification factors (*AF*) of the moisture inflow between 2 and 3^13^,^15^. Each of these scenarios is in agreement with current observations, but they lead to substantially different results after deforestation.

Deforestation in each box of the trajectory is simulated by reducing the total surface heat flux and changing its decomposition into sensible and latent forms: Replacing rainforest by cropland or pasture leads to increased albedo and correspondingly to a reduction of surface net radiation. This results in a decrease of latent heat flux *λ* *E* together with an increase in sensible heat flux *SHF*, such that their sum balances the total change in the surface radiation budget^24^,^25^,^28^. Specifically, due to deforestation, *E* reduces from 0.16 mm/h by at least 20% to 0.13 mm/h, while *SHF* increases by more than 40% (note that in absolute terms, latent heat flux is more than three times higher than sensible heat flux). The precise values of these relative changes are taken from measured differences over southwestern Amazonia^28^, and are consistent with other modelling studies^6^,^9^,^29^. Since deforestation of the Amazon rainforest has been and is projected to remain more intense in the eastern than in the western part of the basin^30^, we always start deforesting in the first, easternmost box of the trajectory, and successively deforest further boxes downstream.

In a first step, we perform single model integrations for deforestation scenarios ranging from zero to hundred deforested boxes (Fig. 3). Deforestation causes a decline of the wind speeds (Fig. 3A), which finally leads to a reduction of *P* to less than 60% at the end of the trajectory for *AF* ≥ 2.5 (Fig. 3B). The onset and slope of the decline depend on the precise value of *E* after deforestation, as well as on the strength of the aforementioned positive feedback, which is expressed in terms of the average total heating over the tropical Atlantic ocean (〈*H*〉^*AO*^) and the amplification factor *AF* (Fig. S6). The rapid decline of *P* is due to the breakdown of the feedback, when the deforestation-induced reduction of water vapour causes the total atmospheric heating over the Amazon to drop below 〈*H*〉^*AO*^. Final values of *P* (i.e., after the breakdown of the feedback) are relatively similar for different values of *E, AF*, and 〈*H*〉^*AO*^ (Fig. S6).

#### Temporal reversibility and stability of solutions

In order to investigate the temporal reversibility of the deforestation process, in a second step we successively deforest boxes from east (box 1) to west (box 100) in single simulation runs for different strengths of the feedback (solid lines in Fig. 3C,D). Thereafter, we invert the process by successively reforesting from west to east (dashed lines in Fig. 3C,D). For moderate amplification factors (*AF* = 2.75), we observe that in this reforestation process, the system first closely follows the curve obtained for deforestation, but then exhibits hysteresis when further boxes upstream are reforested, which implies a considerable delay until the system would recur to its original state. The reason for this behaviour is that the system develops linearly and is thus time-reversible for large numbers of deforested boxes, but develops nonlinearly for less deforested boxes, since the positive feedback is switched on and begins to develop to its original strength in this second regime. Only for the strongest amplification factor consistent with primitive equation models^15^ (*AF* = 3), the process would not be reversible anymore, indicating that even after a complete reforestation, the system could not switch on the positive feedback again. Given that the Amazon rainforest did develop at some point in the past, this suggests that *AF* = 3 might be considered as being too high.

Next, we investigate the stability of these solutions by varying the initial atmospheric moisture *A*(0) and determining the solutions the system will evolve to during the simulation, again for fixed numbers of deforested boxes in each simulation (Fig. 4). There only exists one stable solution for *AF* ≤ 2.25, but for larger amplification factors, there appears a region of bi-stability around 40 deforested boxes: For *AF* = 2.50, we observe a stable solution at high values of *A* between 0 and 50 deforested boxes, and another stable solution with lower values of *A* between 40 and 100 deforested boxes, with an unstable solution connecting the two stable ones between 40 and 50 deforested boxes. This is the signature of a catastrophic fold bifurcation the system undergoes in this regime. For *AF* = 3, the lower stable solution exists for the entire range of deforested boxes, corresponding to the non-reversibility observed in Fig. 3D.

## Discussion

Even under conservative assumptions, our simulations show that when deforestation extends beyond a threshold between 30% and 50% of the total number of boxes spanning the Amazon basin from East to West, precipitation in the remaining, non-deforested parts would rapidly reduce by about 40% for wide ranges of the relevant parameters. The resulting water deficit would be likely to have disastrous consequences in the non-deforested parts of the Amazon basin, where two devastating droughts occurred in the last decade^31^,^32^, but also further downstream of the low-level flow, including the densely populated metropolitan areas of southeastern South America. The latter regions are currently experiencing severe water shortages, however probably due to other hydrological reasons than the moisture inflow from tropical South America^33^,^34^.

As physical mechanism behind this tipping point, we identify the collapse of the positive feedback related to condensational latent heat release over the Amazon rainforest. Sensitively depending on the precise strength of this feedback, the reversibility of this transition by reforestation exhibits pronounced hysteresis. In the conservative spirit of our conceptual approach, we have neglected several additional positive feedbacks existing in this coupled system: First, the direct impact of reduced surface net radiation on convection is neglected due to the persisting problems of adequately parametrising convection even in the most sophisticated climate models^35^. Second, the reduction in downstream *P* will lead to a reduction of *E* also in non-deforested parts of the rainforest, which would, in turn, lead to a further decrease of *P*. Third, impacts on the vertical structure of the atmosphere as well as on the horizontal circulation direction are not considered in our model. On the other hand, a possibly stabilizing feedback, which is induced by changing local circulations due to differential heating between different vegetation types^36^, has also been neglected.

Our conceptual approach clearly indicates the existence of a tipping point in this system and reveals the responsible physical mechanisms. However, the precise values at which this transitions occurs are model-dependent and should therefore, like any other model-derived results, be interpreted with care. Our study thus provides a conceptual basis for further, GCM-based investigations which should specifically focus on the atmospheric mechanisms and critical parameter ranges revealed here.

## Methods

**Figure 1:**
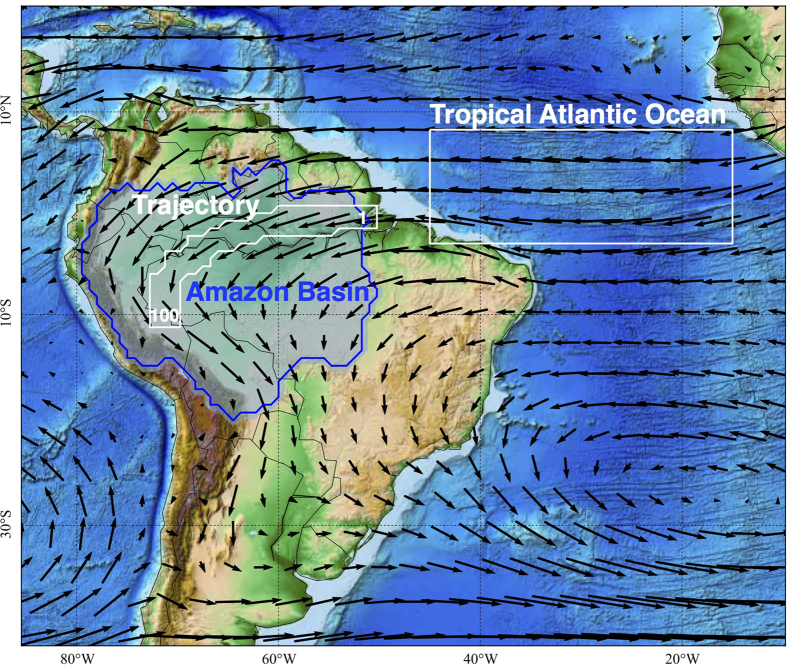
Topography of South America, as well as mean 750 hPa Wind fields for the monsoon season (December–February), as obtained from the ERA Interim reanalysis dataset. The Amazon basin is outlined as the blue area. The trajectory along which we integrate our model is indicated by a white contour line, staring at box #1 to the east, and ending at box #100 to the west. The simplification of considering only this one trajectory, which makes our model one-dimensional, can be justified by the fact that the flow is approximately laminar over the Amazon Basin. The source area of the atmospheric moisture inflow over the tropical Atlantic ocean is indicated by a white box. The gradient of atmospheric heating between ocean and land, *π* = 〈*H*〉^*Trajectory*^ − 〈*H*〉^*AO*^, is quantified by averaging over these two spatial regions (see methods section). The map was created using matplotlib’s basemap toolkit^37^ (http://matplotlib.org/basemap/).

We evaluate the coupled system of equations (1) and (2) along a sequence of 100 boxes 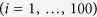, each of length *l* = 30 km, following the trajectory indicated by white contours in Fig. 1 from east to west. The specific equations used for the simulation are


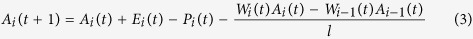



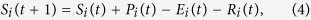


which are integrated in time steps of 1 hour, for a total of 10,000 hours (see Fig. S7) for fixed numbers of deforested boxes. The observables *A* and *S* are given in units of mm of liquid water equivalent, *E, P*, and *R* in units of mm/h, and *W* is given in km/h. Since the integration time step is 1 h, we do not make it explicit in the above discrete equations. Initial conditions and functional dependencies are derived from the ERA Interim reanalysis^26^, confined to the monsoon season (December–February) and the sequence of boxes indicated in Fig. S1. Based on the functional relationships observed in this dataset, *P* is effectively modelled as a linear function of *A* (Fig. S1), while *E* is modelled logistically as a saturation function of *S* (Fig. S2). The values employed here are the monthly means of daily fields, with *P, E*, and *R* rescaled to average hourly rates. We hereby neglect diurnal variations of these variables, because we are merely interested in the average system behaviour on climatological time scales. Several other variables are important for the development of *E*, such as net surface solar radiation, relative humidity, and surface winds. In particular, the apparent decrease of *E* for high values of *S* is due to a shift towards lower values of solar radiation for days with high values of *S*. In addition, *R* is approximated as an exponential function of *S* (Fig. S3).

The Moisture divergence *M* in equation (1) above is defined as the vertical integral of the products of moisture content *A* and wind speed W. Since most of the moisture is in fact concentrated at the lowest atmospheric layers (Fig. S8), we simplify this relationship by only taking into account wind speeds at 750 hPa and transporting the total atmospheric moisture *A* on this single layer. This simplification can be further justified by the fact that 750 hPa wind speeds along the trajectory are very similar to the mean wind speeds averaged from the 700 hPa to the 900 hPa layer (Fig. S9), where the highest wind speeds are found in the ERA Interim data (Fig. S10). Changes in the vertical wind profile due to deforestation are neglected in this setting.

Wind speeds *W* are comprised of a prescribed, constant trade wind component *W*^*trade*^ and a dynamical component *W*^*H*^ corresponding to the acceleration due to the atmospheric heating gradient between ocean and land. The first component (*W*^*trade*^) is modelled as a shifted sigmoid function of the box number *i* in order to take into account the blocking effect of the Andes mountain range at the western boundary of the Amazon basin:


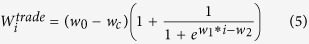


Based on the assumption that the gradient in atmospheric heating will mainly enhance the flow in the eastern parts of the basin, the dynamical component *W*^*H*^ is modelled as


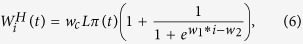


where *π*(*t*) denotes the difference between total atmospheric heating (*H* = *H*^*sensible*^ + *H*^*latent*^ + *H*^*radiative*^) averaged over the trajectory across the Amazon basin, and over the spatial box over the tropical Atlantic ocean (both shown in Fig. 1), respectively: *π* = 〈*H*〉^*Trajectory*^ − 〈*H*〉^*AO*^, where 〈·〉^*R*^ indicates the spatial average over a region *R* (see Fig. S11 for a spatial plot of total atmospheric heating as derived from ERA Interim). The average heating over the spatial box named Tropical Atlantic Ocean is 〈*H*〉^*AO*^ = 97 W/m^2^, and we present our simulation results for 〈*H*〉^*AO*^ = 90 W/m^2^, 〈*H*〉^*AO*^ = 95 W/m^2^, and 〈*H*〉^*AO*^ = 100 W/m^2^ in order to account for the uncertainties involved in the data themselves, as well in choosing this spatial box (see Fig. S7). If *π* becomes negative, we put 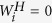, indicating a breakdown of the feedback.

**Figure 2:**
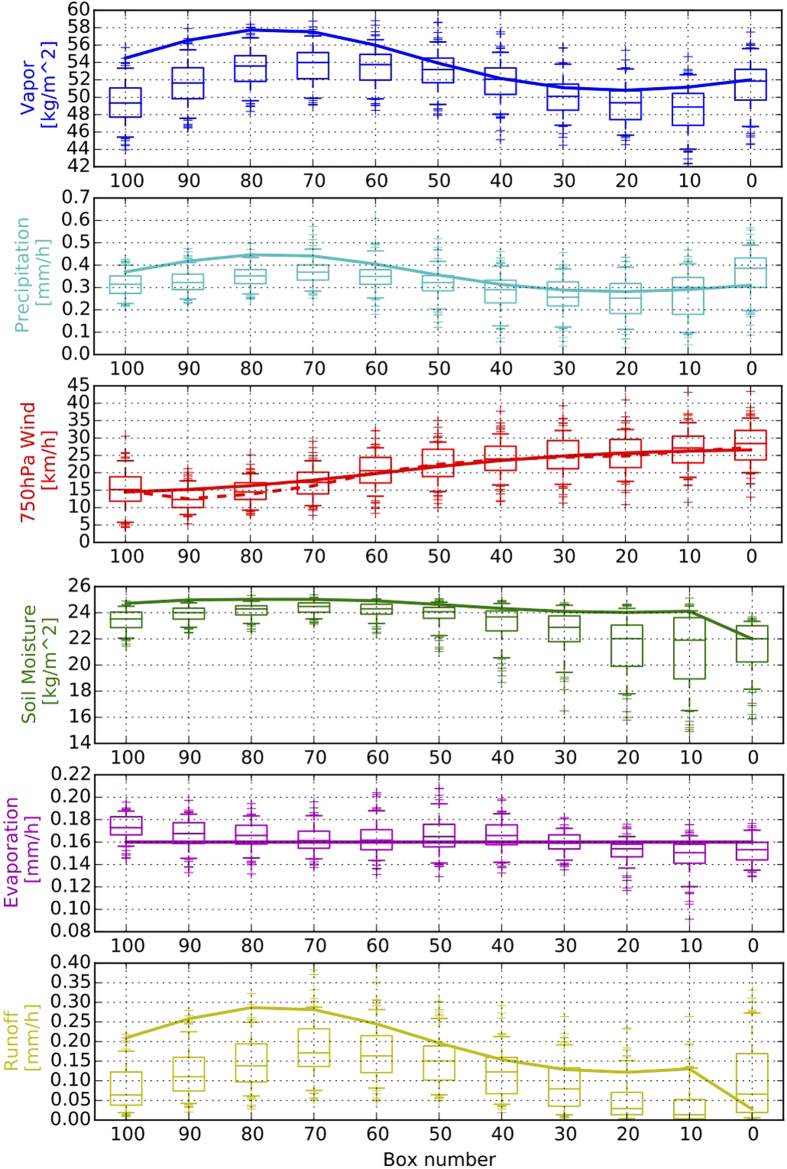
Comparison between ERA interim reanalysis values (box plots) and model results (solid lines) along the trajectory indicated by a white contour line in Fig. 1. Mean values of atmospheric vapour content *A* (blue), precipitation *P* (cyan), wind speeds at 750 hPa *W* (red) soil moisture *S* (green), evapotranspiration *E* (magenta), and river runoff *R* (yellow) are shown. For the wind speeds, we also show the mean across atmospheric layers from 700 hPa to 900 hPa as obtained from ERA Interim (red dashed line). The box plots should be read as follows: Each box box extends from the lower to upper quartile of the corresponding values, with a line at the median. The whiskers (horizontal lines below and above the boxes) indicate the 5th and 95th percentiles. All values beyond the whiskers are considered as outliers, and plotted individually.

**Figure 3:**
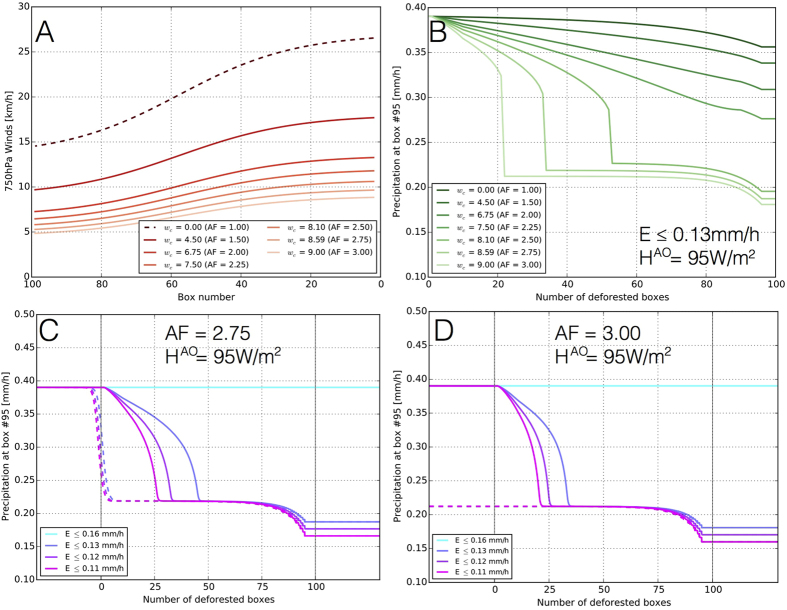
Consequences of deforestation on the low-level circulation and precipitation. (**A**) Impact of different choices of *w*_*c*_, corresponding to different amplification factors *AF* as indicated in the legend, on the 750 hPa wind speeds along the trajectory shown in Fig. 1. The dashed line shows the total wind speed *W* = *W*^*trade*^ + *W*^*H*^, which is equal for all choices of *w*_*c*_, while the solid lines show only the first component *W*^*trade*^ (see methods section for details). (**B**) Dependance of precipitation *P* in box 95 (located in the western Amazon) on the number of deforested boxes (*E* ≤ 0.13 mm/h) for different amplification factors as indicated in the legend. (**C**) Reversibility of the deforestation process for *AF* = 2.75. We present the results for different strengths of deforestation (*E* ≤ 0.13 mm/h to *E* ≤ 0.11 mm/h) to show that they are relatively stable against small variations of the upper bound on *E*. The solid (dashed) lines indicate the deforestation (reforestation) process and should be read from left (right) to right (left). Deforestation is initiated at 5000 h (left vertical line) and completed at 15000 h (right vertical line) in the deforestation simulation, while reforestation is initiated at the right vertical line and completed at the left vertical line. Note that the process is reversible with significant temporal delay due to hysteresis. The development for no deforestation (*E* ≤ 0.16 mm/h) is shown for comparison. (**D**) Same as (**C**), but for *AF* = 3.00. Note that for this strength of the positive feedback induced by the heating gradient between ocean and land, the process would not be revertible anymore. For (**C**,**D**) only, the model is integrated for 20,000 h, in order to allow the system to adapt to changing numbers of deforested boxes in single simulation runs. Note that the label “box number” in *A* indicates the position along the box trajectory, while the label “Number of deforested boxes” in the remaining panels indicates how many boxes are deforested, starting from the easternmost box (#1).

**Figure 4:**
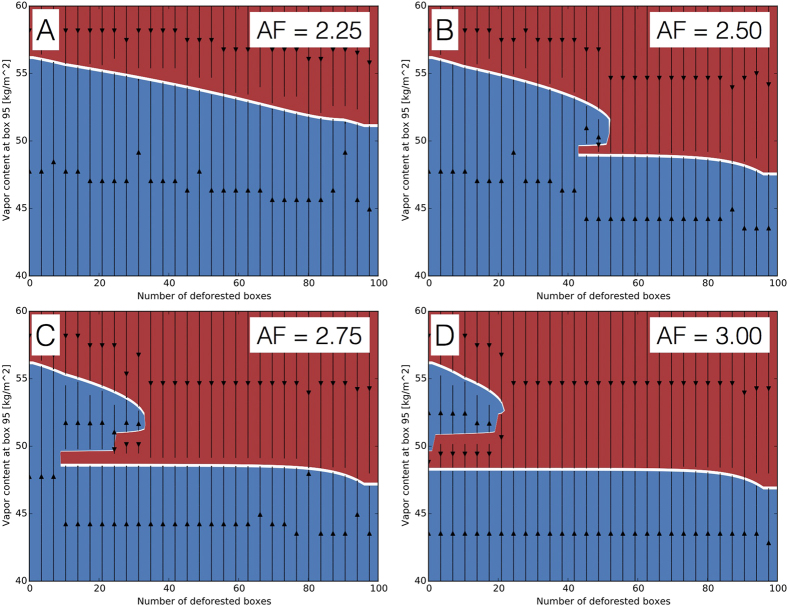
Stability diagram for the solutions obtained from simulations obtained for *E* ≤ 0.13 mm/h, 〈*H*〉^*AO*^ = 95 W/m^2^, and amplification factors *AF* = 2.25 (**A**), *AF* = 2.50 (**B**), *AF* = 2.75 (**C**), and *AF* = 3.00 (**D**). The arrows indicate the evolution of atmospheric moisture *A* from a given initial condition to the next stable solution, which are indicated by thick white lines. In contrast, the arrows point away from unstable solutions, indicated by thin white lines. Red (blue) colours indicate regions where the atmospheric moisture *A* decreases (increases) to the next stable solution.

The dimensionality factor *L* as well as the parameters *w*_0_, *w*_1_, *w*_2_ are adjusted such that the modelled wind speeds approximately meet the observed ones (Fig. 2). For our simulation, we choose *w*_0_ = 13.5, *w*_1_ = 0.06, *w*_2_ = 3.4, and *L* = 1/*π*(0), which corresponds to the heating gradient for the undisturbed case of no deforestation. Our results are insensitive to varying *L, w*_0_, *w*_1_, and *w*_2_ within ranges that still assure the resulting total wind speed to be equal to the observed ones. In contrast, the consequences of deforestation are highly sensitive to variations of *w*_*c*_ between 0 and *w*_0_, although before deforestation the same total wind speed *W* is obtained in all cases. Physically, varying *w*_*c*_ corresponds to controlling the strength of the heating-related feedback and its contribution to wind speeds along the trajectory, while keeping the total wind speeds constant: For *w*_*c*_ = 0, there would be no contribution from the heating gradient (*AF* = 0), whereas for *w*_*c*_ = *w*_0_, the entire inflow would be due to *LH* release. For *w*_*c*_ = 6.75, we obtain an amplification of the inflow to South America by a factor of *AF* = 2, whereas for *w*_*c*_ = 9.00, the inflow is enhanced by a factor of *AF* = 3 (see also Fig. 3A). Choices of *w*_*c*_ within this range meet the conditions found for this geographical region using a primitive equation model^13^,^15^.

By adding independent white noise increments of standard deviation *σ* = 0.1 to each *A*_*i*_(*t* + 1) in equation (3), we are able to show that our results are stable against small levels of noise (Fig. S12).

## Additional Information

**How to cite this article**: Boers, N. *et al*. A deforestation-induced tipping point for the South American monsoon system. *Sci. Rep.* **7**, 41489; doi: 10.1038/srep41489 (2017).

**Publisher's note:** Springer Nature remains neutral with regard to jurisdictional claims in published maps and institutional affiliations.

## Supplementary Material

Supplementary Information
