# Supplementary material for: A deforestation-induced tipping point for the South American monsoon system
Source: Sci Rep. 2017 Jan 25;7:41489. doi: 10.1038/srep41489 (PMC5264177; doi:10.1038/srep41489)
Supplement: Supplementary Information [file srep41489-s1.pdf]

Supplementary Information for manuscript “A deforestation-induced tipping point for the South American monsoon system”

Niklas Boers[1,2,*], Norbert Marwan[2], Henrique M.J. Barbosa[3], and Jürgen Kurths[2,4,5,6]

[1]Ecole Normale Supérieure, Geosciences Department and Laboratoire de Météorologie Dynamique, Paris, F-75230, France
[2]Potsdam Institute for Climate Impact Research, Potsdam, 14473, Germany
[3]University of São Paulo, Institute of Physics, São Paulo, 05508-090, Brazil
[4]Humboldt University, Department of Physics, Berlin, 12489, Germany
[5]Nizhny Novgorod State University, Department of Control Theory, Nizhny Novgorod, 603950, Russia
[6]University of Aberdeen, Institute for Complex Systems and Mathematical Biology, Aberdeen, AB24 3UE, United Kingdom
[*]boers@pik-potsdam.de

ABSTRACT

The supplementary information contains 12 supplementary figures.

References

1. Hunter, J. D. Matplotlib: A 2D graphics environment. *Computing in Science and Engineering* **9**, 99–104 (2007). 0402594v3.

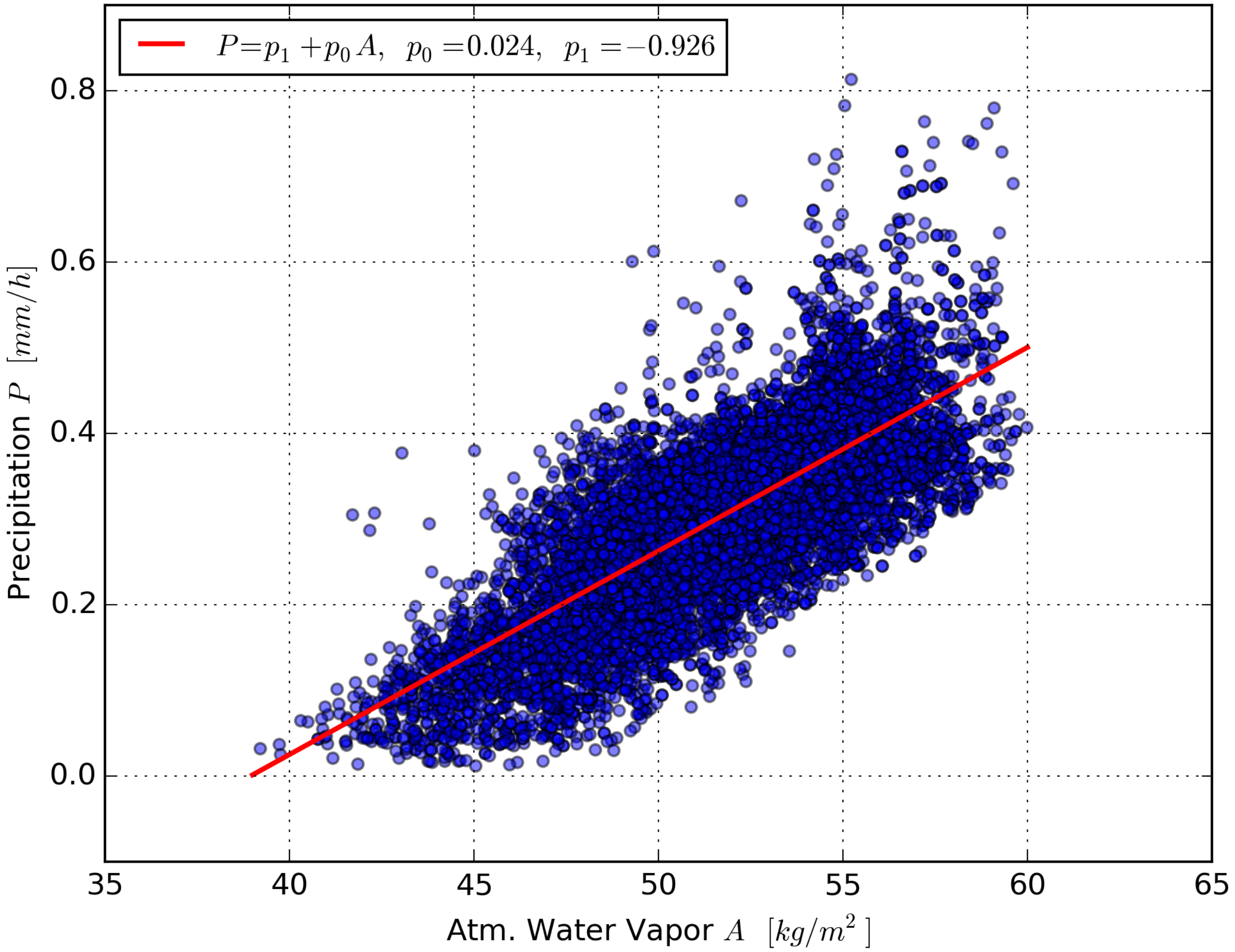

Figure S1: Scatter plot of precipitation P versus atmospheric moisture A, and linear best fit based on ordinary least squares minimisation. The goodness of fit is estimated using a χ^2 test, yielding a p-value $p < 10^{-3}$.

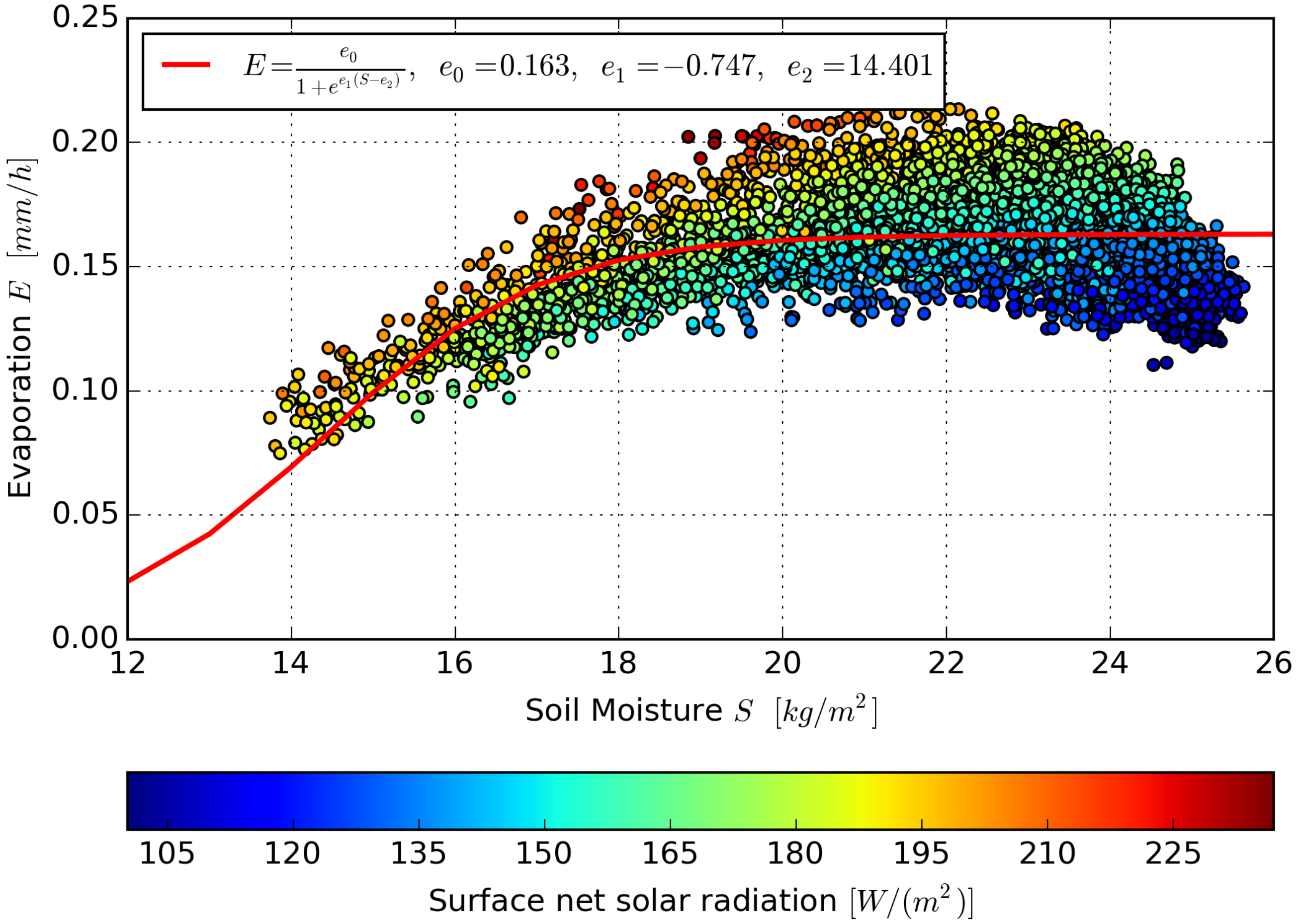

Figure S2: Scatter plot of evapotranspiration E versus soil moisture S, and logistic best fit based on ordinary least squares minimisation. The goodness of fit is estimates using a χ^2 test, yielding a p-value $p < 10^{-3}$. The colormap indicates the dependance of E on net surface radiation, which causes the apparent decrease of E for high values of S.

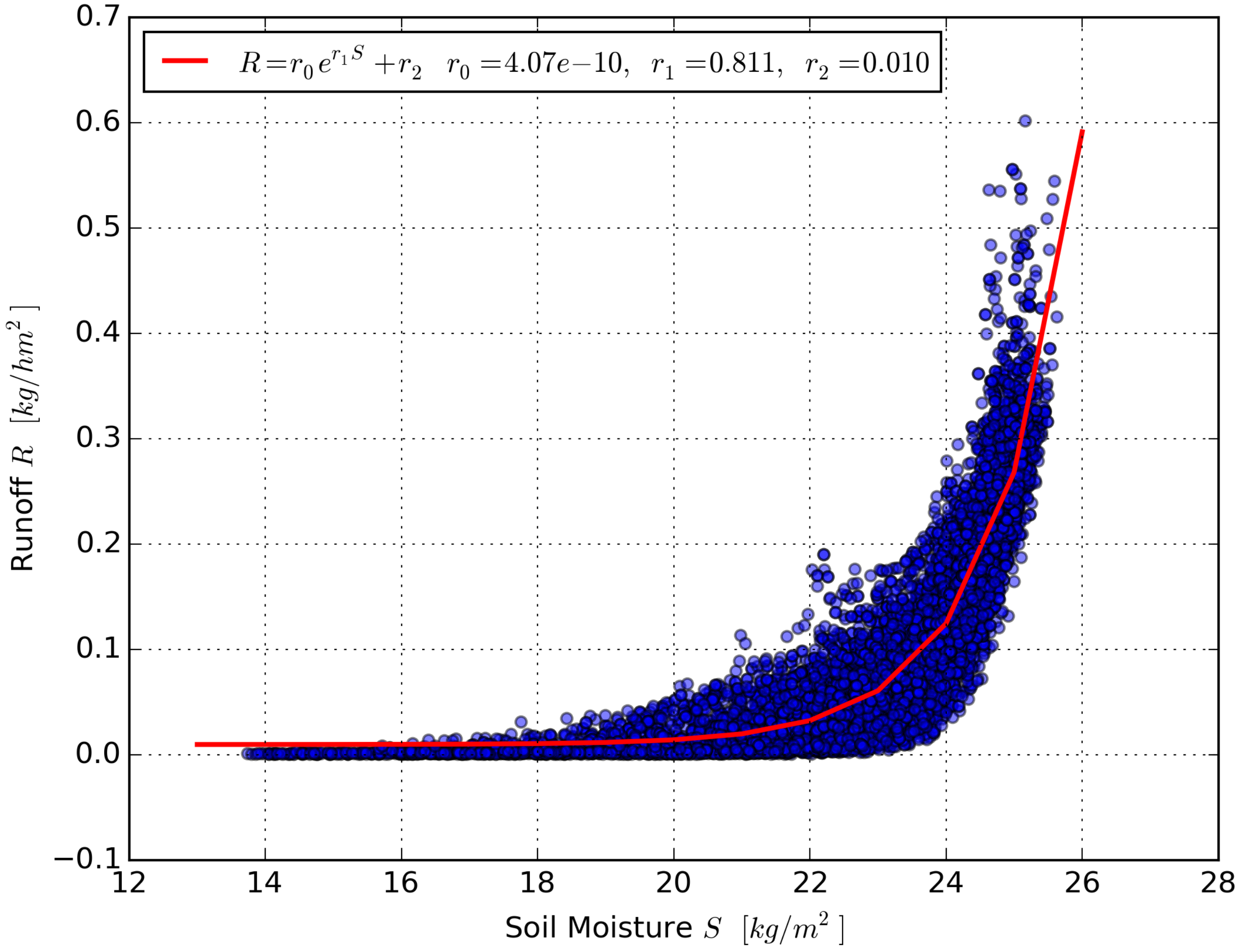

Figure S3: Scatter plot of runoff R versus soil moisture A, and exponential best fit based on ordinary least squares minimisation. The goodness of fit is estimates using a χ^2 test, yielding a p-value $p < 10^{-3}$.

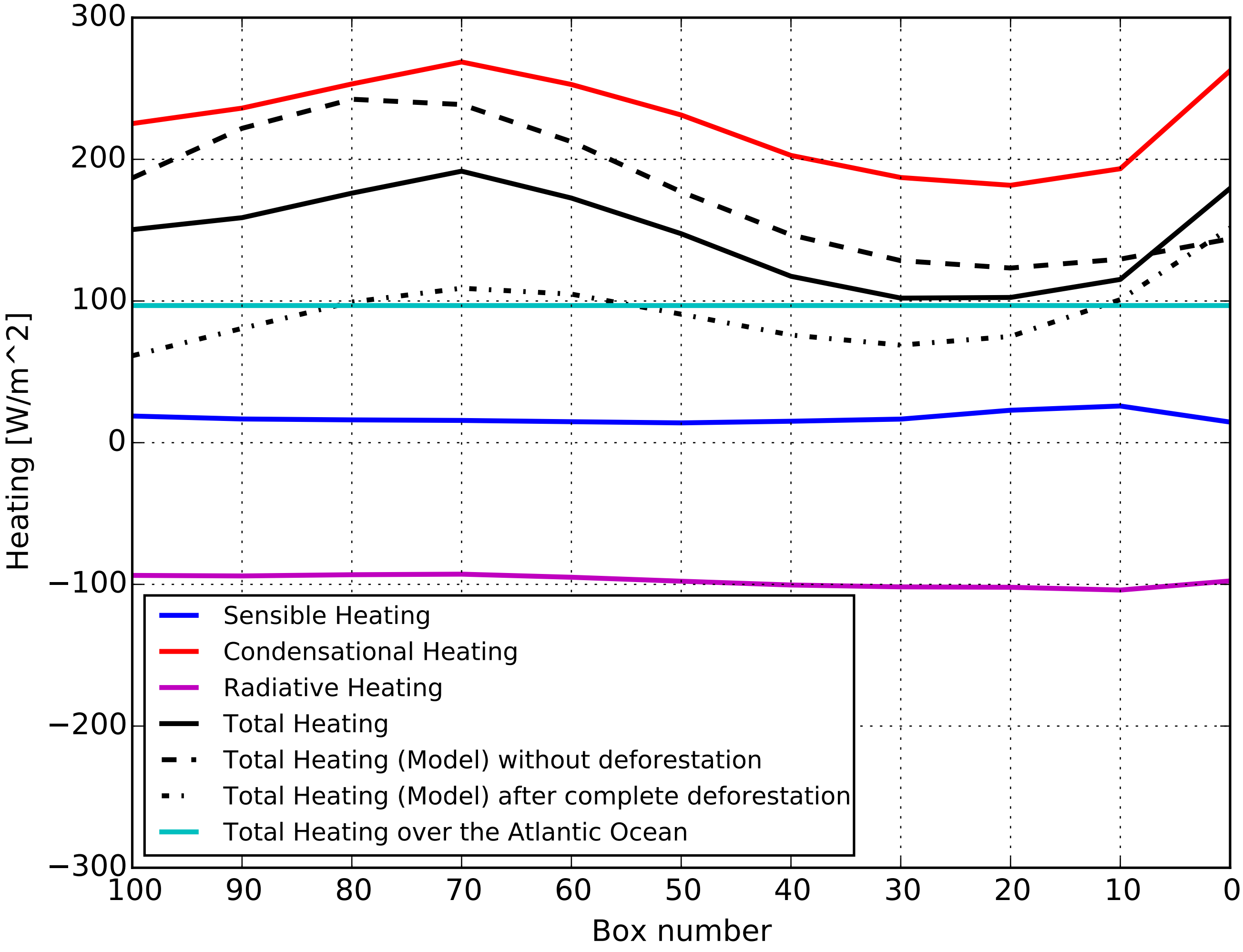

Figure S4: Components of atmospheric heating and their sum $H = H^{sensible} + H^{latent} + H^{radiative}$ as derived from ERA Interim (solid black line), as well as the total heating resulting from our model (dashed black line). For comparison, also the simulated total heating H after complete deforestation (total 100 boxes) for $AF = 2.50$ and $E \leq 0.13$ mm/h (dot-dashed black line), as well as the mean heating over the tropical Atlantic ocean $\langle H \rangle^{AO}$ are shown.

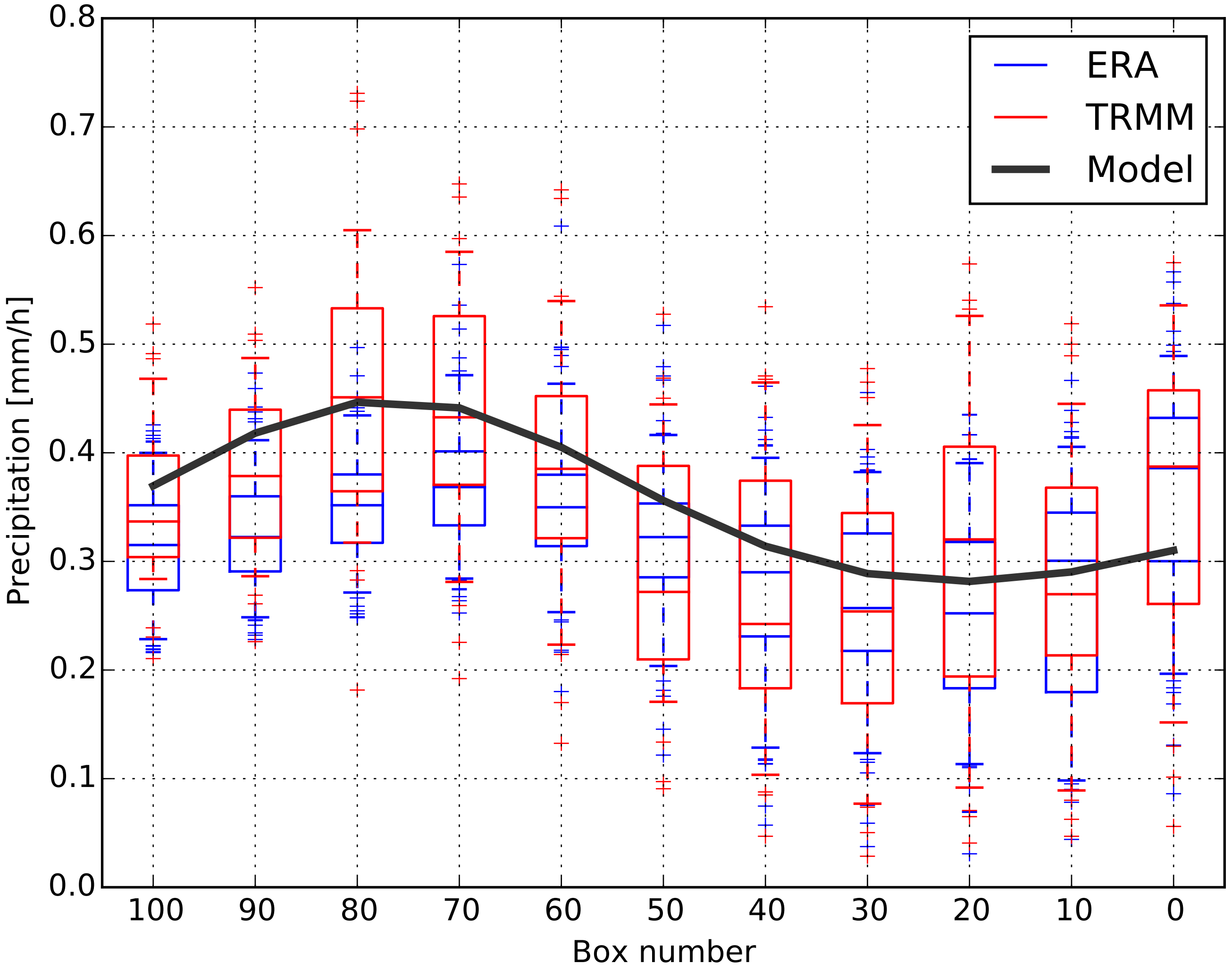

Figure S5: Comparison between precipitation values obtained from ERA interim (blue box plots), TRMM 3b42 V7 (red box plots) and our conceptual model (black solid line) along the trajectory indicated by a white contour line in Fig. 1 in the main text.

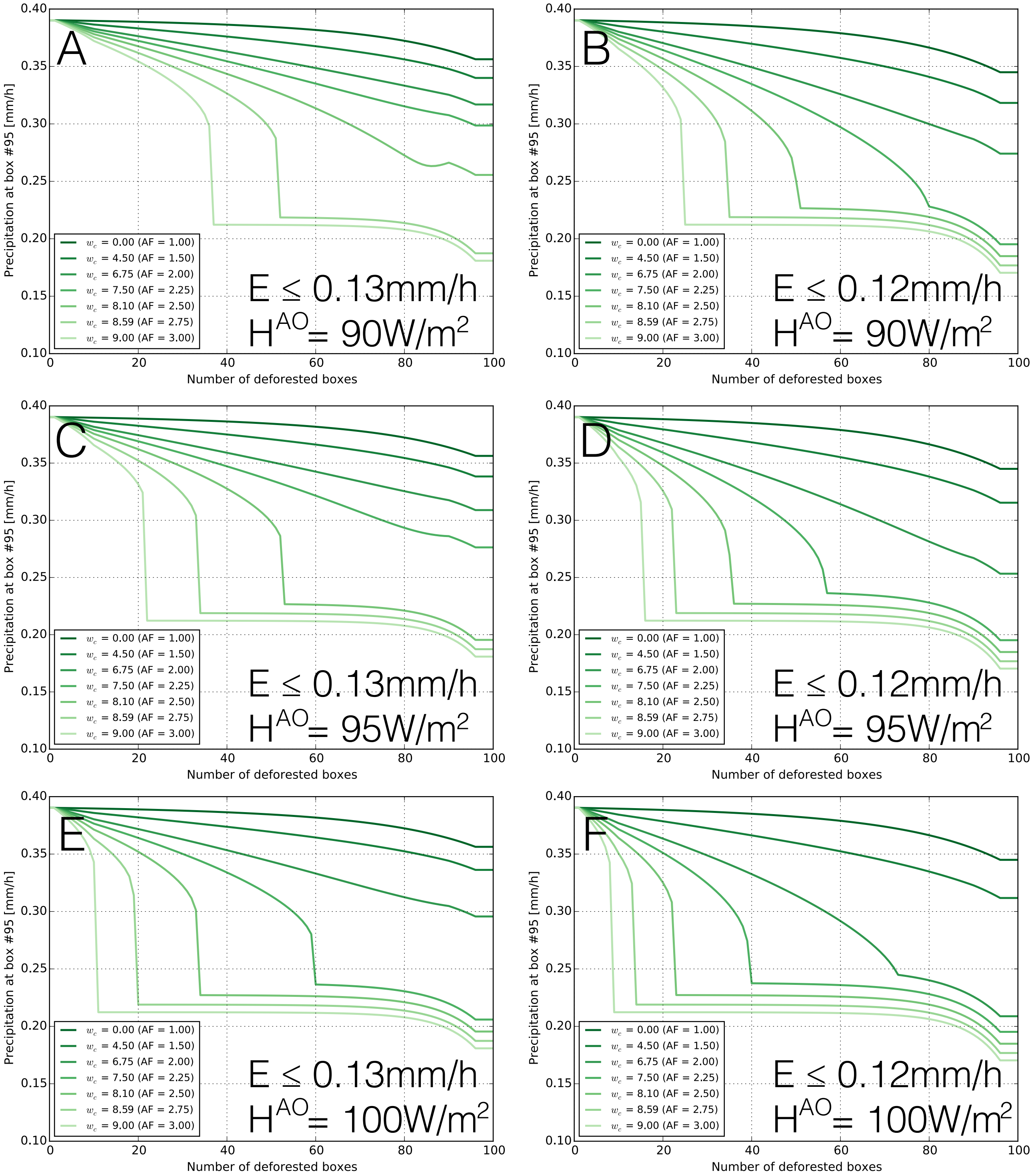

Figure S6: Same as Fig. 3B in the main text for $E \leq 0.13$ mm/h (left column) and $E \leq 0.12$ mm/h (right column), and different choices of total heating over the tropical Atlantic ocean, as used to compute the heating gradient $\pi = \langle H \rangle^{Trajectory} - \langle H \rangle^{AO}$ between ocean and land: $\langle H \rangle^{AO} = 90\,\mathrm{W/m^2}$ (top row), $\langle H \rangle^{AO} = 95\,\mathrm{W/m^2}$ (middle row), $\langle H \rangle^{AO} = 100\,\mathrm{W/m^2}$ (bottom row). Note that the transition in the precipitation regime occurs earlier and more pronounced for stronger impacts of deforestation on evaporation ($E \leq 0.12$ mm/h), but also for higher values of atmospheric heating over the tropical Atlantic ocean $\langle H \rangle^{AO}$, which correspond to smaller heating gradients π. In (A), e.g., a small increase of P around 90 deforested boxes can be observed, which is due to the increase of sensible heat flux caused by deforestation (see also Fig. S7).

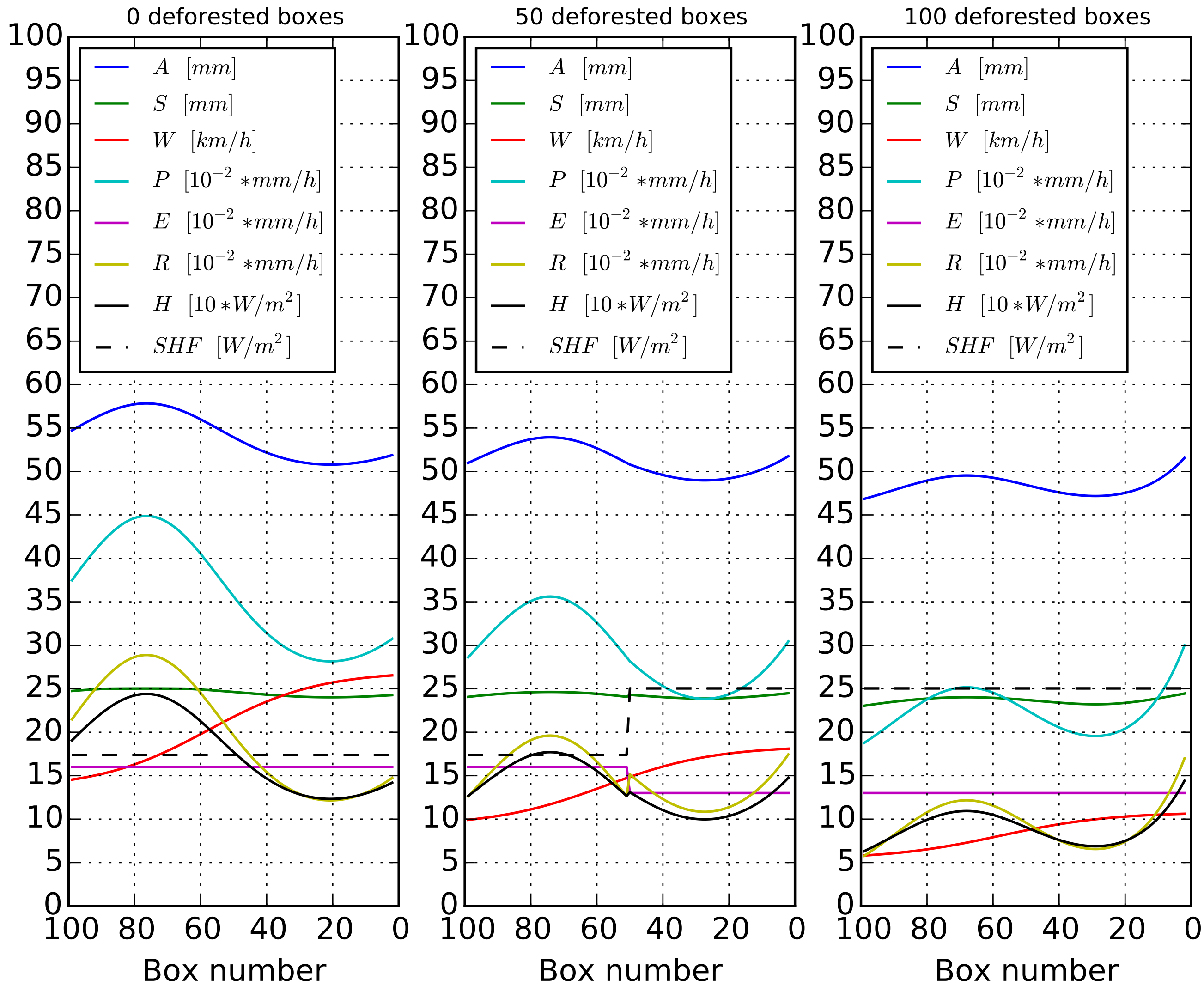

Figure S7: Simulated development of the variables A, S, W, P, E, R, total atmospheric heating H, and sensible heat flux SHF along the trajectory shown in Fig. 1 in the main text, for $E \leq 0.13$ mm/h after deforestation, $AF = 2.50$, and $\langle H \rangle^{AO} = 95\,\mathrm{W/m^2}$. Variables are rescaled as indicated in the legend, such that they can all be shown in a single figure for comparison. All values along the trajectory are shown for 0 deforested boxes (left), 50 deforested boxes (middle), and 100 deforested boxes (right). Note that soil moisture S varies very little along the trajectory, leading also to small variations in E before deforestation. In addition, note the decrease of E and simultaneous increase of SHF after deforestation.

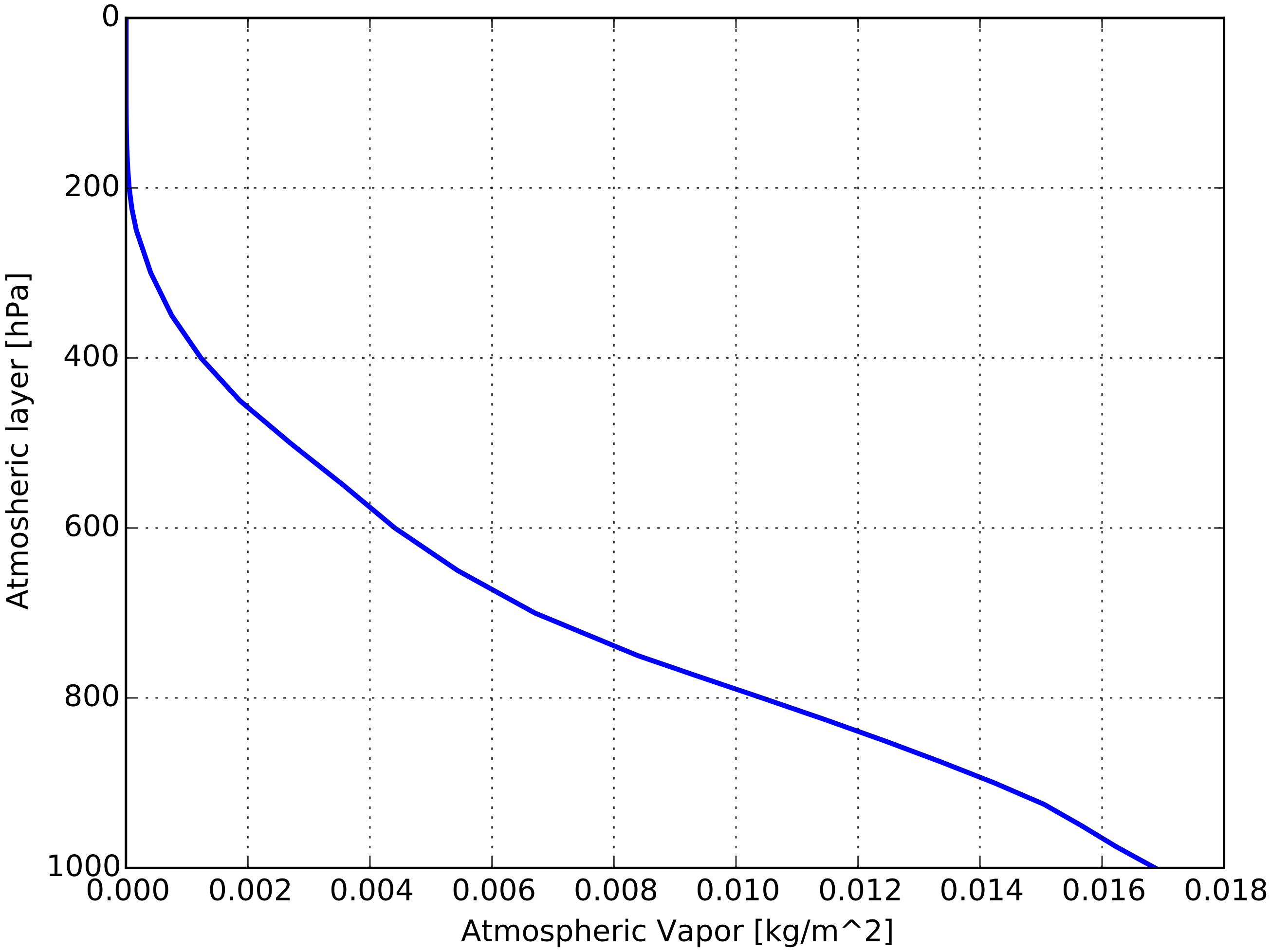

Figure S8: The distribution of atmospheric moisture content over the vertical atmospheric levels, as obtained from the ERA Interim dataset.

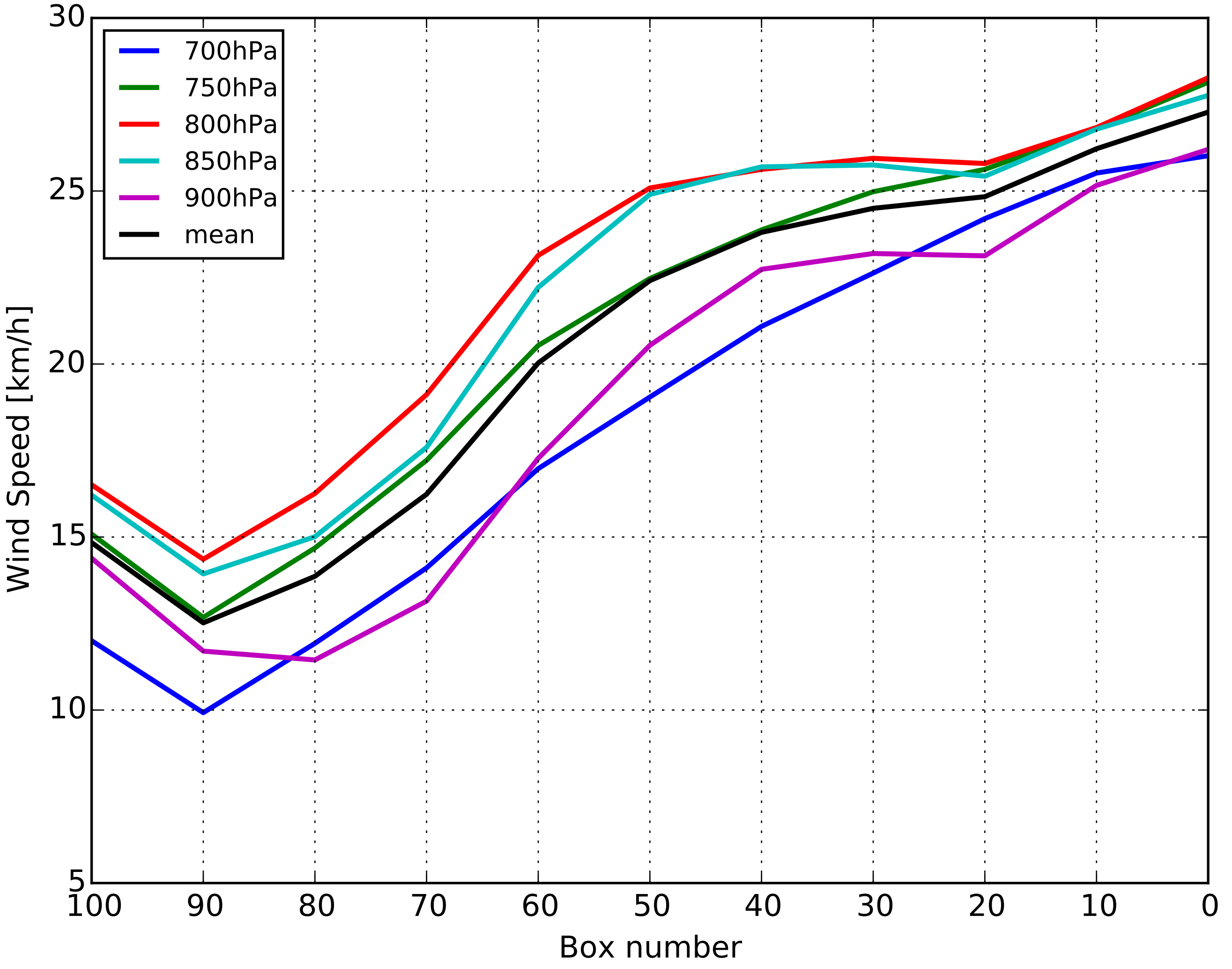

Figure S9: Mean wind speeds W along the trajectory shown in Fig. 1 in the main text at different vertical levels as indicated in the figure legend. Note the great similarity between the mean over all vertical levels between 700 hPa and 900 hPa and wind speeds at the 750 hPa layer. Values are taken from the ERA Interim reanalysis data.

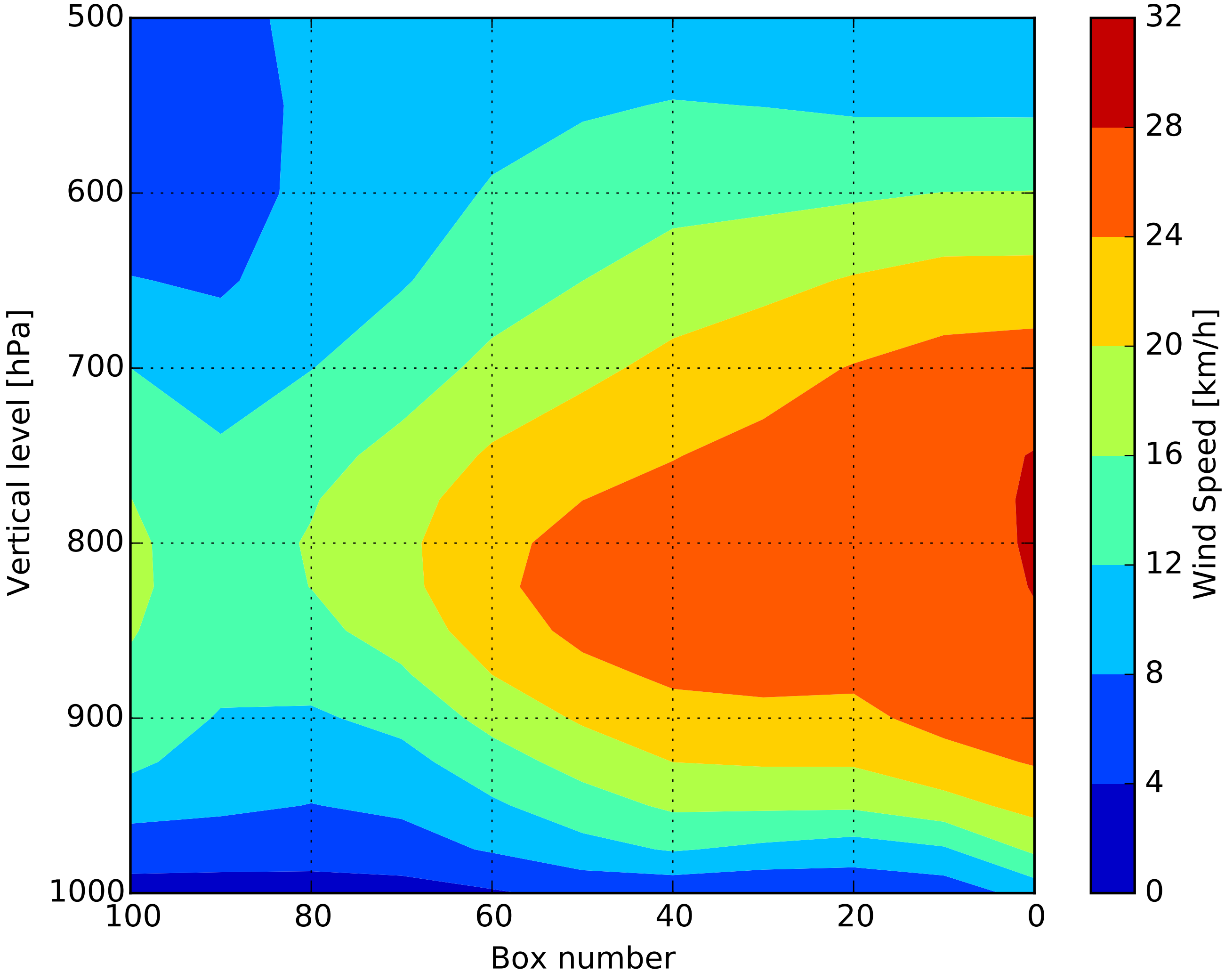

Figure S10: Vertical wind profile along the trajectory shown in Fig. 1 in the main text, from 1000 hPa to 500 hPa. Note that wind speeds are highest between 700 hPa and 900 hPa.

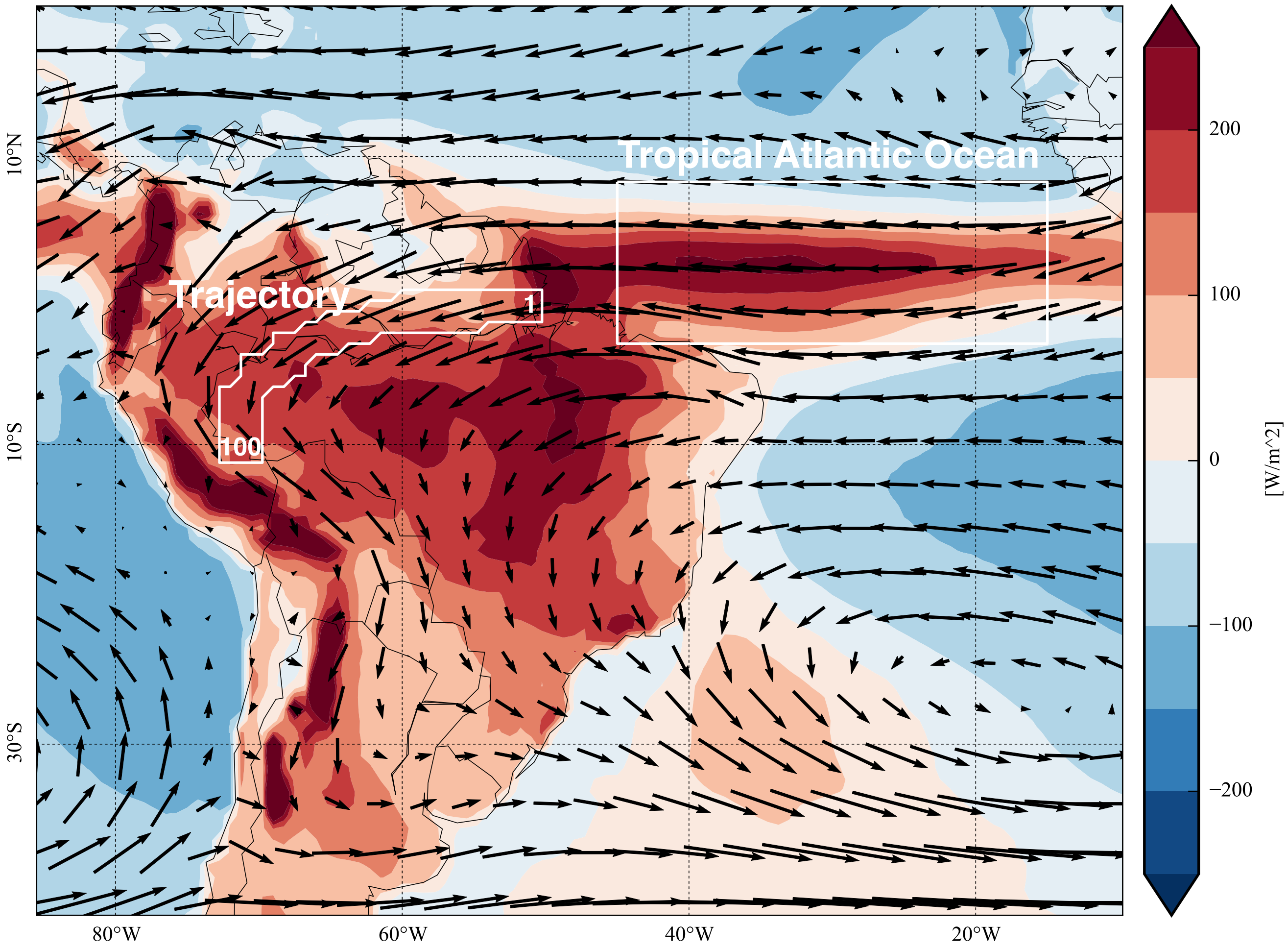

Figure S11: Total atmospheric heating over the considered spatial domain, obtained from the ERA Interim dataset. The average heating over the spatial box named Tropical Atlantic Ocean here is $\langle H \rangle^{AO} = 97\,\mathrm{W/m^2}$, and in order to account for the involved uncertainties, we therefore show our simulation results for $\langle H \rangle^{AO} = 90\,\mathrm{W/m^2}$, $\langle H \rangle^{AO} = 95\,\mathrm{W/m^2}$, and $\langle H \rangle^{AO} = 100\,\mathrm{W/m^2}$ (see Fig. S7). The map was created using matplotlib's basemap toolkit[1] (http://matplotlib.org/basemap/).

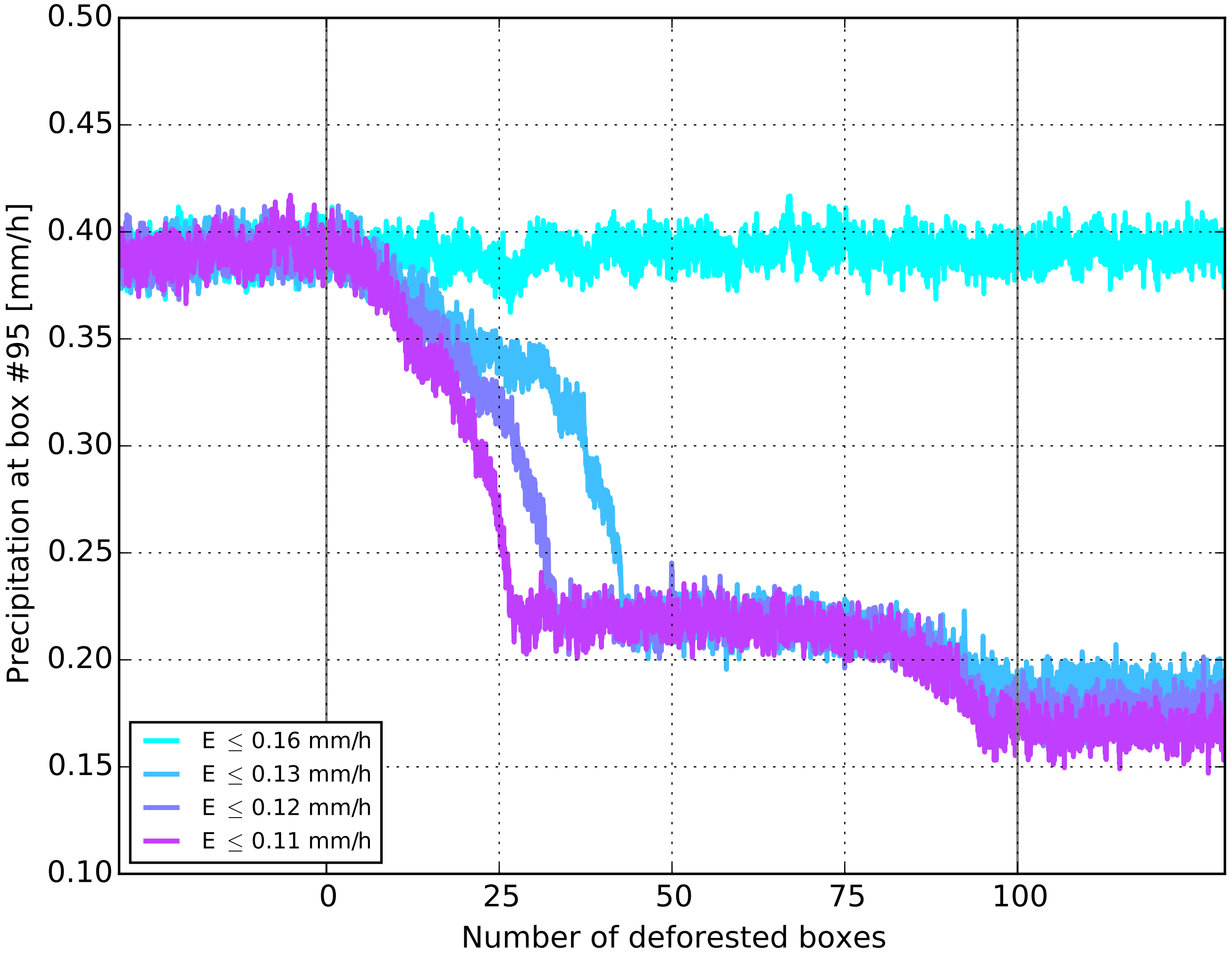

Figure S12: Same as Fig. 3C in the main text, but for small levels of additive white noise ($\sigma = 0.1$) in each time step and at each box. Only the deforestation (solid lines in Fig. 3C) is shown.
